# Hierarchies of description enable understanding of cognitive phenomena in terms of neuron activity

**DOI:** 10.1007/s10339-024-01181-5

**Published:** 2024-03-14

**Authors:** L. Andrew Coward

**Affiliations:** College of Engineering, Computing and Cybernetics, Australian National University, Canberra, Australia

**Keywords:** Cognitive phenomena, Anatomical structures, Neuron activity, Hierarchies of description

## Abstract

One objective of neuroscience is to understand a wide range of specific cognitive processes in terms of neuron activity. The huge amount of observational data about the brain makes achieving this objective challenging. Different models on different levels of detail provide some insight, but the relationship between models on different levels is not clear. Complex computing systems with trillions of components like transistors are fully understood in the sense that system features can be precisely related to transistor activity. Such understanding could not involve a designer simultaneously thinking about the ongoing activity of all the components active in the course of carrying out some system feature. Brain modeling approaches like dynamical systems are inadequate to support understanding of computing systems, because their use relies on approximations like treating all components as more or less identical. Understanding computing systems needs a much more sophisticated use of approximation, involving creation of hierarchies of description in which the higher levels are more approximate, with effective translation between different levels in the hierarchy made possible by using the same general types of information processes on every level. These types are instruction and data read/write. There are no direct resemblances between computers and brains, but natural selection pressures have resulted in brain resources being organized into modular hierarchies and in the existence of two general types of information processes called condition definition/detection and behavioral recommendation. As a result, it is possible to create hierarchies of description linking cognitive phenomena to neuron activity, analogous with but qualitatively different from the hierarchies of description used to understand computing systems. An intuitively satisfying understanding of cognitive processes in terms of more detailed brain activity is then possible.

## Introduction

A human brain is made up of around 86 billion interconnected neurons. Each neuron has a complex internal structure, and has inputs via synapses from an average of about 10 thousand other neurons. There are thus of the order of 10^15^ synapses in the brain. The most complex computing systems can have of the order of 10^15^ interconnected transistors. The way in which the network of transistors results in the features of the computing system is understood, in the sense that the network has been designed by human engineers to implement the desired features, and can be modified to implement desired feature changes. Although there are no resemblances between transistor networks and neuron networks, a key question is whether the techniques used to understand electronic systems can be applied in any way to understanding brains.

One objective of neuroscience is to understand cognitive processes in terms of the activity of neurons and their components (Swanson and Lichtman 2016). Modern techniques allow the acquisition of huge amounts of data about the brain, including connectivity and dynamic imaging (Misic and Sporns 2016). The challenge is to extract understanding of the brain from the vast amounts of data (Swanson and Lichtman 2016). These data have been used to support large-scale data mining efforts such as the Human Brain Project (Markram 2006) and the Human Connectome (Leergaard et al. 2012). However, use of the data in these ways has led to very limited progress in conceptual and theoretical understanding of the brain (Fregnac 2017).

It was originally argued by Marr (1982) that some intermediate computational levels of description are required to achieve understanding. Network neuroscience has built on this idea, proposing multiple different levels of description from metabolic pathways through neuron circuit dynamics to whole brain functional networks (Bassett and Sporns 2017). However, the need for descriptions on different levels to be related to each other in a detailed but comprehensible fashion is rarely addressed. It has even been argued that understanding will require models (or descriptions) on different levels of detail that coexist with no clear relationship between them, each explaining different features of the data (Churchland and Abbott 2016).

There have been extensive efforts to model large-scale brain activity using nonlinear dynamical systems theory (Breakspear 2017). Such models involve approximating neuron activity in different ways, in the extreme some models assume that the exact states of individual neurons are irrelevant. Although dynamical systems models have had some successes in modeling macroscopic phenomena such as seizures (Breakspear et al. 2006)*,* anesthesia (Bojak and Liley 2005), and EEG frequencies (Freyer et al. 2011), no ability to model individual cognitive processes has been demonstrated. Furthermore, to many neuroscientists, an approach that discards much of the cell- and circuit-specific neuroscience information is not attractive.

Then there is the question of what exactly “understanding” would be (Coward 2013; Bertolero and Bassett 2020). Ideally, it must be possible to organize all the data in a way that relates synapse and neuron activity to specific cognitive processes in an intuitively satisfying manner. It is clear that, caricaturing the situation somewhat, such understanding cannot involve a neuroscientist simultaneously imagining the activity of all the neurons participating in a cognitive process. Some degree of approximation will be needed.

Computing systems are well understood, in the sense that the performance of specific features can ultimately be understood in terms of transistor activity. The techniques used to study the brain as a system have been used to try to understand a microprocessor, including measuring how each transistor is connected to all the others, lesioning single transistors to determine the effects on feature operations, plotting the spike rate of individual transistors as a function of some measurable response (such as the luminance of the most recently displayed screen pixel), and analyzing the average transistor activity within localized regions. It was found that these techniques did not lead to the type of understanding of the processor that most students of electrical engineering obtain (Jonas and Kording 2017).

In the case of a computing system, there are some key factors that make understanding possible. There are multiple levels of description, and most important it is possible to map between these levels. The higher levels of description are more approximate, but the approximations are carefully managed. To provide a degree of understanding of the brain comparable with that which can be achieved for a computing system, these same factors must be present. Approaches like dynamic systems theory use a much more simplistic approach to approximation and do not support mapping between multiple levels of description from cognitive features to detailed neuron activities. As a result, these approaches will not lead to effective understanding of cognition in terms of neuron activity.

The key question is whether the techniques used to understand computing systems can be modified to be applied to the brain. Natural selection pressures have resulted in the organization of physical brain resources in such a way that it is possible to apply techniques analogous with those used to understand computing systems. This approach is radically different from conventional computational modeling, but can lead to an intuitively satisfying understanding of cognitive processes in terms of the activity of anatomical structures, neurons, and the constituents of neurons.

## Standard brain computational modeling approaches

In physical sciences like quantum mechanics or fluid dynamics, observed phenomena reflect collective behavior and not that of individual units. Mathematical laws governing macroscopic variables are capable of explaining and predicting such observed phenomena. Dynamical systems theory starts from the premise that brain phenomena can be explained and predicted on the basis of the collective behavior of neurons rather than their individual activity. Although implementation details differ, these models use differential equations for groups of spiking neurons. These groups, also called ensembles or populations, are defined by coupling parameters representing synaptic interactions between neurons that tend to promote synchronization within the group. There is also a stochastic term modeling fluctuations attributed to random neuron activity. These fluctuations tend to disrupt synchronization (Breakspear 2017). In some cases, the interactions between several separate populations of neurons are modeled (Breakspear et al. 2004). Essentially, the approximation made by these models is that neurons within an ensemble can be regarded as identical. A wide range of electroencephalogram (EEG) phenomena have been successfully modeled with this approach, including fluctuations in the alpha rhythm (Freyer et al. 2011), changes to EEG during seizures (Breakspear et al 2006), and during general anesthesia (Bojak and Liley 2005). In addition, irregular brain activity following oxygen deprivation has been modeled (Roberts et al. 2014), and the brain activity of various brainstem nuclei during the sleep–wake cycle (Phillips and Robinson 2007). However, none of the successfully modeled phenomena are specific cognitive processes.

### Comparison with computing systems

In a computing system, there are similarities between different individual transistors, but each transistor is connected with a different group of transistors and other components. This connectivity is precisely specified by a designer, and if a single connectivity error is made, there will be a problem in the performance of user features. The operation of the features thus depends on the unique connectivity pattern of the transistors. In other words, for the purposes of understanding the operation of user features, transistors cannot be regarded as identical. Use of differential equations would depend on the assumption that transistors are identical, and such equations are never used to design the features of a computing system. However, it is possible to use differential equations to determine overall system characteristics like heat flow, or electrical noise that can interfere with other systems. For these types of characteristics, the approximation that components are all more or less identical is appropriate. Such characteristics are analogous with overall brain activation states as studied by EEG measurements.

## What can be learned about brains by studying computing systems?

As discussed earlier, the techniques generally used to study brains have little value for understanding computers (Jonas and Kording 2017). However, can the way we understand computing systems provide insight into a way to achieve understanding of brains? Brains are nothing like computers, and it would be deeply misleading to directly use the way a computer performs its functions as a model for how a brain operates. Nevertheless, the way we go about understanding the designs of complex real-time computing systems has some critical lessons for how to go about understanding brains (Coward 2001; 2013). In computers, there are multiple intermediate levels of description, analogous with those postulated as being necessary for understanding brains, but with the essential feature that it is possible to map (or translate) fairly precisely between the different levels. This mapping is made possible in computing systems by the use of just two types of information process on every level: the instruction and the data read/write. Within the hierarchy of descriptions, there is sophisticated management of the use of approximation, with higher levels more approximate, detailed levels more precise, far removed from the very general approximation that all transistors are identical.

How can this apply to the brain? Although there are no direct resemblances between complex computing systems and brains, there are a couple of what might be called metasimilarities. One metasimilarity is that both types of system are made up of huge numbers of interconnected information processing components, where the performance of system features depends on the unique connectivity environment of each component. A second metasimilarity is that both types of systems experience some practical pressures that severely constrain their architectures. These practical pressures include the need to limit the amount of information processing resources and the need to change or add features or behaviors in such a way that other features are not affected. Resource constraints force the organization of information processing resources into modular hierarchies. The need to achieve an adequate compromise between limiting resources and modifiability constrains the highest level architectures into one of just two forms (Coward 2001). If features are defined under external intellectual control (i.e., by a designer), the system is constrained into the familiar memory-processing-input/output form sometimes called the instruction or von Neumann architecture. If features are defined heuristically (i.e., learned), the system is constrained into a form called the recommendation architecture. All information processes in the instruction architecture are one of two general types: the instruction and the data read/write. All information processes in the recommendation architecture are also of two general types: the condition define/detect and the behavioral recommendation (Coward 1990). Although the information processes in the two architectures are qualitatively different, in both cases the existence of the two types is critical for understanding the features of the system in terms of component activity (Coward 2013).

### Both computing systems and brains are networks of uniquely interconnected components

The first metasimilarity is that both types of system have huge numbers of components and a complex network of connectivity between them. The most complex computing systems have multiple trillions of components like transistors, capacitors, resistors, and a few other types. The transistors form a network in which each transistor is connected with a different group of other transistors. No two transistors are connected in exactly the same way to exactly the same group of other components, so each transistor has a unique connectivity environment within the system as a whole. Most important, it is this unique connectivity that makes the system features work. The pattern of connectivity between transistors must be precisely specified by a designer, and a single connectivity error will mean a system malfunction.

In the human brain, there are about 86 billion neurons and hundreds of trillions of synapses between them. Each neuron is connected with a different group of other neurons, and successful completion of cognitive tasks depends on this connectivity. In the case of brains, the connectivity is initially defined genetically but from there is largely evolved by subsequent experience. As discussed earlier, such a dependence of cognitive features on unique individual neuron connectivity makes dynamic system modeling of cognitive features impossible, although general brain activity such as that revealed in EEG measurements is possible.

### Both computing systems and brains are control systems that associate information conditions with behaviors

Both brains and computers are control systems. They get information about their environment and about their own internal state. They process this information to detect specific circumstances, or conditions, and in response to conditions perform appropriate behaviors. For example, suppose that when a document is being typed, a computer gets information that the shift key and the A key have been pressed at the same time. It also has information that a specific document is open in a window on the screen, the cursor is at a specific location in the document, and a particular font is associated with that location. The combination of all these information conditions is associated with the behavior of displaying a particular form of the letter "A" at a specific location on the screen.

When a person sees a dog, their brain gets information derived from the dog, its position and behavior; the general circumstances such as the presence of other people; and propioceptic information about the position of the body and the state of muscles. The combination of all these information conditions could be associated with the behavior of verbally warning a companion about a potentially aggressive dog.

The critical difference between the two types of system is that in a computer the conditions, behaviors, and associations between them are all specified in advance by a designer. In a brain, most of the conditions, behaviors, and associations between them are defined heuristically.

### Both computing systems and brains are subject to practical considerations that constrain their general architectures

The third metasimilarity is that for both computing systems and brains, there are some practical pressures on the way information processing resources are organized (Coward 2001). These pressures can be labeled resource constraints, modifiability, repairability, and constructability.

In the case of computing systems, although individual transistors are cheap, a poor design could result in such large numbers being required that the system cost would be excessive. Features are regularly added or modified but there is always a tendency for such changes to introduce undesirable side effects on other features. There is a practical need to limit these side effects as much as possible. If some component or structure in a computing system is damaged, it must be possible to repair the system by identifying and replacing the defective structure. Manufacturing processes that have a relatively low chance of errors that introduce defects will result in much lower cost systems. All these considerations tend to constrain the form of the system architecture, and as the ratio of number of features to available resources increases, the constraints become tighter.

Natural selection exerts some analogous pressures on brains. It two species need to learn similar behaviors, but the brain of one species requires fewer resources (like neurons), that species will have a natural selection advantage. If the brain of one species can learn new behaviors with limited effect on the performance of already learned behaviors, again that species will have a natural selection advantage over a species in which new learning often disrupted earlier learning. A brain that can recover to some degree from damage will have a natural selection advantage over a brain for which damage is almost invariably fatal. A brain with a development process that has a relatively low chance of error will have a natural selection advantage over a brain for which the development process is more error prone. For brains, there is another aspect of the modifiability constraint. Evolution occurs through the occurrence of mutations to DNA, some of which have performance advantages. However, any mutation may also have disadvantages. If all mutations had fatal consequences, evolution in response to changes in the environment would be impossible. Hence there is a natural selection advantage to brains in which mutations to DNA are not necessarily fatal.

These constraints are in conflict. To give a simple example, features require information processing resources, and a feature change will require some changes to information processing resources. If the information processing resources for every feature were physically separate, any feature could be modified without side effects on any other feature. However, separate resources for every feature would result in a need for vast amounts of resources. Sharing of resources across different features is essential. Hence the need to conserve resources is in conflict with the need to avoid undesirable side effects as a result of changes. It can be demonstrated that for any system which performs a complex combination of features, the compromises required to satisfy these conflicting pressures result in the highest level architecture being constrained into one of two possible forms (Coward 2001; 2013).

In the case of computing systems, information processing resources tend to be constrained into a general form called the instruction (or von Neumann) architecture. All the information processes within this architecture are of one of just two general types: instructions and data read/writes. The architecture contains processor modules that mainly perform instruction processes, memory modules that mainly perform data read/write processes, and input/output modules that perform mixtures of these processes.

In the case of systems that need to learn a complex combination of behaviors, it can be demonstrated that information processing resources tend to be constrained into a general form called the recommendation architecture (Coward 2001; 2013). All the information processes within this architecture are also one of just two general types: condition define/detects and behavioral recommendations. These two types are qualitatively different from the information processes in the instruction architecture (Coward 1990). Brain architectures tend to contain modules that specialize in condition define/detect processes (the cortex in mammals), modules that specialize in behavioral recommendation processes (basal ganglia and hippocampus for different types of behavior), and special purpose modules that implement the selected behaviors (the thalamus), manage prioritization of behavioral types (amygdala and hypothalamus), and speed up the implementation of previously learned sequences of behaviors (cerebellum) (Coward 2011). This anatomical architecture can be observed across all species with brains, from mammals to birds (Jarvis et al 2005), and all the way to cephalopods like the octopus (Shigeno et al. 2018). Note that although this high level architecture is universal, at more detailed levels natural selection can result in different physiological implementations of the processes performed by the high level anatomical modules (Pryluk et al. 2019).

A fundamental difference between the two architectures is that in the instruction architecture the detection of an information condition is associated with a command (i.e., an instruction) to perform some behavior. In the recommendation architecture, the detection of a condition is associated with a range of recommendations with different weights in favor of different behaviors.

## Resource organization in computing systems

In a computing system, the resources that perform information processing are organized into modular hierarchies (Bourne et al 1980; Keiser and Strange 1985). A major system is divided up into frames which are the highest level modules. Frames are made up of printed circuit assemblies. Printed circuit assemblies are made up of integrated circuits and other components. Integrated circuits are subdivided into areas, areas into sections, sections into the most detailed modules which are logic gates and other functions. Groups of transistors make up these most detailed modules. The reason for this organization is to reduce the total information processing resources required. Any user feature requires many information processes. Generally there are similarities between some of the information processes required by different features. To economize on resources, all the information processes are divided into groups on the basis of similarity. Very detailed modules made up of small groups of transistors perform groups of very similar processes. The transistors and connectivity of these most detailed modules are customized to carry out the processes as efficiently as possible. Groups of very detailed modules with some degree of process similarity make up somewhat higher level modules, the similarity making a further degree of resource sharing possible. This resource sharing essentially defines the somewhat higher level module. Groups of these somewhat higher level modules with a somewhat less degree of process similarity make up even higher level modules, enabling further resource sharing. This continues until no useful resource economy can be achieved by going to a yet higher level.

Modules thus exist to reduce the total amount of processing resources required by the system by sharing resources for similar processes (Coward 2001). Such resource sharing requires information exchange between modules. An important feature of a modular hierarchy is that this required information exchange is minimized as far as possible. There are two reasons for this. One reason is that the more a module depends upon information from other modules to carry out its processes, the more time the module will have to spend just waiting for that information, slowing down the system operations. The second reason is that to implement a change to a feature, some modules have to change. The changes may result in undesirable side effects on other features supported by the same modules, and further changes must be made to correct for the side effects. Sometimes a change will also change the information generated by those modules, which would change the operations of other modules that use that information. Those operation changes could result in further undesirable side effects on different features that use those other modules. The greater the information exchange, the greater the propagation of undesirable side effects following feature changes (Kamel 1987). A major driving force on system architecture is the need to achieve an adequate compromise between the resource sharing benefit and the undesirable side effects of changes associated with information exchange.

In computing systems, there are in fact two fairly separate modular hierarchies, for hardware and software (Lasker 1979; Soni et al. 1995). It is easier to make changes using software, but the problem of changes tending to introduce undesirable side effects remains.

It is important to note that because modules are defined by groups of similar information processes, there are no simple correspondences between modules and system features (Coward 2001). Any one feature will require processes performed by many different modules, and any one module will provide information processes in support of many different features.

### How computing systems are understood

Using differential equations to understand how complex physical systems change over time requires the use of approximations. As described earlier, either the system must be regarded as made up of just a few components, or large numbers of identical components. Designing a computing system requires a much more sophisticated use of approximation (Coward 2013). The two factors that make possible the approximations needed for understanding are the organization of information processing resources into a modular hierarchy and the existence of just two information processing types: instructions and data read/writes (Coward 1990).

As an example, a personal computer contains a small number of high level modules like CPU, RAM, monitor interface, keyboard interface, wireless interface etc. These modules are printed circuit assemblies inside the computer. The end-to-end operation of a feature can be described in terms of the interactions between these modules (Coward 2021). The information content of such a description is limited, so a human being can follow it. However, such a description is approximate. To make it more precise, it is necessary to think about the interactions between the submodules of each module, like the integrated circuits. Such a description is more accurate, but involves a lot more information. So a designer could not simultaneously imagine the end-to-end operation of a feature at this level of detail. Only a small part of the feature operation could be thought about at the same time. But even this level of description is still approximate, and to get even more accuracy, it is necessary to shift to a description in terms of the submodules of the integrated circuits: different sections in its layout. A designer can only think about an even smaller part of feature operation at this level at the same time. For yet more accuracy, you need a description in terms of cells within sections. More accuracy still requires descriptions in terms of even smaller units like logic gates within cells. For very precise descriptions, it is necessary to think about the interactions between individual transistors within a logic gate, and beyond that, thinking about individual transistors in terms of quantum mechanics may be needed. For a designer to understand how some detailed parts work together, it is always necessary to think about those parts on the next higher level of description.

So understanding requires what could be called a hierarchy of descriptions (Coward 2013). The system behavior can described on many different levels of detail. The higher level descriptions have lower information content, but are more approximate. The more detailed descriptions are more precise, but have much higher information content. However, fairly precise translation between levels is possible. Given this hierarchy, the system can be understood for the purposes of designing it or modifying it. Such understanding involves approximate thinking about large chunks of system operation at high level, more accurate thinking about smaller chunks at intermediate levels, and precise thinking about tiny chunks of system operation at very detailed levels. To understand how the detailed chunks fit together, it is always necessary to move to a higher level of description. At every level, the information needed to think about a chunk of the system operation is within human mental bandwidth, and system understanding requires constant shifting between levels.

For a hierarchy of descriptions like this to exist, hardware must be organized in a hierarchy of modules with much more information exchange within a module than between peer modules (Coward 2001). Higher level descriptions are in terms of the interactions between higher level modules, ignoring the interactions within those modules. It must also be fairly easy to map (or translate) precisely back and forth between descriptions on different levels. Translation between languages is difficult because a concept in one language may not exactly correspond with any concept in the other language. In some situations, multiple translations are possible, and none of them is really adequate. This problem is avoided in electronic design because descriptions on every level are expressed using the same two information processing concepts. At a detailed level, all information processes are instructions or data read/writes. At higher levels, processes are instructions or data read/writes made up of combinations and sequences of more detailed instructions and data read/writes. This consistency makes it straightforward to map between levels.

## Resource organization in the brain

In the case of brains, natural selection pressures in favor of efficient use of resources have also resulted in a hierarchy of modules. In such a hierarchy, modules specialize in different types of information processes and there is much more information exchange within a module than between the module and its peer modules. The highest level modules of the brain include the cortex, thalamus, basal ganglia, hippocampal system, amygdala, hypothalamus, basal forebrain, and cerebellum (Coward 2013). The degree of connectivity can be used as a proxy for degree of information exchange. There is much more connectivity within the cortex than between the cortex and other brain structures. The cortex is divided up into about 150 area modules. An area is made up of column modules. A column is made up of pyramidal neurons. A pyramidal neuron has a number of terminal dendritic branches, and each branch has a number of synapses from other neurons. There is much more connectivity within an area than between areas. There is much more connectivity within a column than between columns. There is much more connectivity (in this case by chemical interactions) within a pyramidal neuron than between neurons. There is much more interaction within a terminal dendritic branch than between branches. A similar pattern of connectivity can be observed in the subcortical structures. Analogous to the situation in computers, there are no correspondences between modules on any level and cognitive features or categories. Any cognitive feature requires processes performed by many modules, and any module supports many different cognitive features. For example, episodic memory requires processes performed by multiple cortical areas, the thalamus, basal ganglia, basal forebrain, hippocampus, hypothalamus, amygdala, and cerebellum (Dickerson et al. 2010; Andreasen et al 1999; Scimeca et al. 2012).

### How the brain can be understood

Natural selection pressures on brains drive both the organization of information processing resources into modular hierarchies and the existence of just two types of information processes. This means that the prerequisites are present in the brain for creating hierarchies of description analogous with (but qualitatively different from) the hierarchies that make detailed understanding of complex computing systems possible.

In the brain, all information processes are of one of just two types: condition definition/detection and behavioral recommendation (Coward 1990). At the highest level, a cognitive process can be described by combinations and sequences of these information processes (Coward 2013; 2021). The information processes at this highest level are made up of combinations and sequences of information processes of these same two types in major anatomical structures. The information processes in major anatomical structures are made up of combinations and sequences of information processes of these same two types in more detailed anatomical structures, and so on down to information processes implemented by chemistry within neurons.

Such hierarchies of description make possible an intuitively satisfying understanding of cognition in terms of anatomy and physiology. As an illustration, the way in which such hierarchies of description work for one type of cognitive process is described in the next section.

## Understanding the creation and recall of episodic memories

To understand how hierarchies of description work to relate a cognitive phenomenon to neuron activity in the brain, the example of learning and subsequently recalling the memory of a specific event will be used. Such recall requires activation of a population of neuron conditions (also called neuron receptive fields) similar to the population active during the original event, in the absence of the sensory inputs present during that event. Indirect activations in the absence of sensory inputs can only use information that is currently available to neurons. There are four types of such information. Neurons can be indirectly activated on the basis of recent simultaneous activity; on the basis of frequent past simultaneous activity; on the basis of past simultaneous activity at a time when significant condition changes were occurring; or on the basis of current simultaneous activity. These four mechanisms access recommendation strengths not available from receptive fields directly detected in current sensory inputs. These mechanisms support, respectively, priming, semantic, episodic, and working memory (Coward 2013). However, any actual cognitive task will generally require a combination of these mechanisms (Coward 2013). As a first step in understanding episodic memory, the information processes required will be described at the highest and the most detailed levels.

### Information processes at high level

**Fig. 1:**
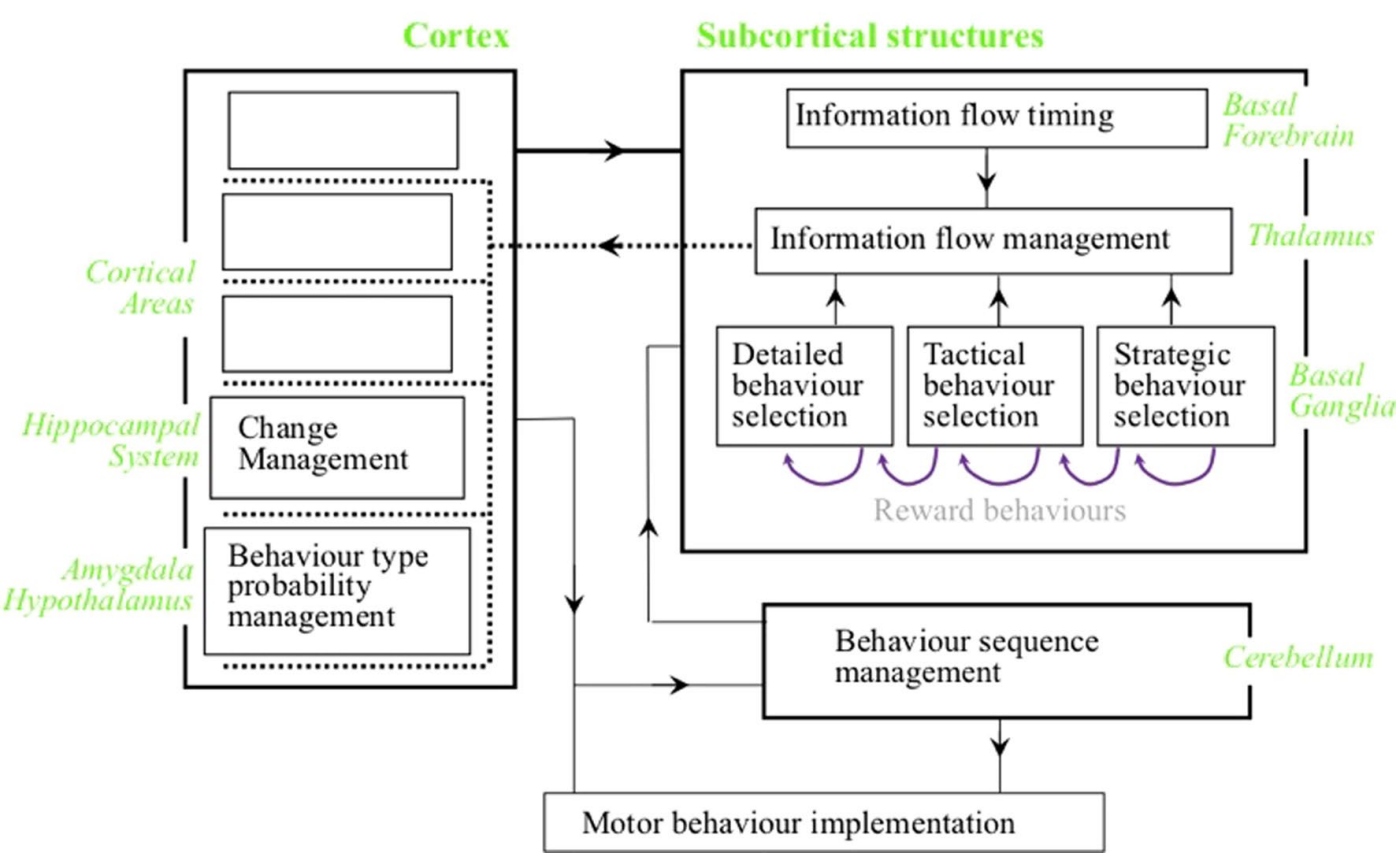
Recommendation architecture of the brain at the highest level. The cortex defines and detects conditions, with different areas defining conditions on different levels of complexity. Some condition detections are communicated to the subcortical structures, where each condition is associated with a range of recommendations in favor of different behaviors, each with an individual weight. Different cortical areas are most effective for recommending different types of behaviors. The subcortical structures determine the behaviors most strongly recommended across all currently detected conditions, and implement those behaviors. Sequences of behaviors often used in the past are recorded in the cerebellum. Such sequences are selected once as a whole by the basal ganglia, then implemented rapidly by the cerebellum with no requirement for the basal ganglia to select each behavior individually. All the different anatomical structures can be clearly identified on the basis of different neuron types, different patterns of connectivity including much more connectivity within a structure that between structures, and physical separation from other structures by regions with relatively few neuron central bodies (Coward 2013)

The highest level architecture of the brain is illustrated in Fig. 1, showing the major anatomical structures and the types of information processes performed. The cortex defines and detects conditions within information derived from the senses and the internal state of the brain itself. The basal ganglia interpret each current condition detection received from the cortex as a range of recommendations in favor of different behaviors, each with an individual weight. The basal ganglia implements the behaviors with the largest total current weight. Many of the behaviors are releases of information into the cortex from the senses, between cortical areas, or out of the cortex to drive muscle movements. Once selected by the basal ganglia, these release behaviors are implemented by the thalamus. Other behaviors make changes to recently utilized recommendation weights (i.e., reward behaviors). These reward behaviors are also recommended by cortical condition detections but the basal ganglia implements these behaviors on itself.

Because each condition recommends a wide range of behaviors, any changes to a condition can jeopardize the integrity of those recommendation weights. Such changes must therefore be carefully managed, and this management is the primary role of the hippocampal system (Coward 2010). Conditions detected in three cortical areas associated with the hippocampus recommend changes, and changes are selected on this basis by the hippocampus proper. A cortical column defines and detects a group of relatively similar conditions (Tanaka 2003), and a primary role of columns is to generate the information needed to perform change management (Coward and Gedeon 2015). The hippocampus proper selects the groups of cortical columns most appropriate for expansions, and these selections are mapped back through the hippocampal areas.

The amygdala and hypothalamus recommend and implement general types of behavior. Selections of general types of behavior are implemented by release of the conditions that recommended them to cortical areas effective for recommending more specific behaviors.

Selection of behavior by the basal ganglia takes a certain amount of time. For sequences of behaviors that often need to be carried out in the same order, this time can be reduced by recording the sequence in the cerebellum. The sequence is selected once as a whole by the basal ganglia, then rapidly executed by the cerebellum with no further reference to the basal ganglia.

### Information processes at a detailed level

At the most detailed level, both condition definitions and behavioral recommendation weights are implemented by the strengths of glutamatergic synapses (Coward 2013). A cortical pyramidal neuron fires in response to any combination of inputs from other pyramidal neurons with total synaptic weight above a threshold. Its receptive field (or condition) is therefore defined by the weights of all the synapses and how they are combined across terminal dendritic branches. Individual medium spiny neurons (MSNs) in the striatum of the basal ganglia each correspond with one behavior. The weight of a synapse from a cortical pyramidal neuron on to an MSN is the recommendation weight of the pyramidal neuron receptive field in favor of the corresponding behavior.

Most changes to a pyramidal neuron receptive field are slight expansions in the range of circumstances in which the receptive field is detected. When a pyramidal neuron fires, there is a slight increase in the weights of all its recently active glutamatergic synapses. These increases gradually decay back to their original values. However, other pyramidal neurons have recommendation strength in favor of a long-term prolongation of recent increases. If this behavior is accepted, it is implemented by the firing of dopaminergic neurons that prolongs recent increases. More major expansions are driven by the hippocampal system. In order to have adequate ranges of recommendation strengths available, at least a minimum number of conditions must be detected. The cortical areas associated with the hippocampal system recommend expansions. If the circumstances have some degree of novelty, the number of cortical receptive field detections will be below the minimum. In this situation, the hippocampus proper selects and drives appropriate expansions. The hippocampal system achieves this by sending additional inputs to the selected pyramidal neurons, causing them to fire in circumstances more distant from their programmed condition, resulting in a larger than usual expansion in their receptive field definition (Coward 2011).

Long-term changes to recommendation weights occur by similar dopaminergic mechanisms (Coward 2013). If a behavior is accepted, MSNs corresponding with that behavior fire strongly. This firing temporarily increases the weights of all the active synapses on the MSN. Some cortical pyramidal neurons have recommendation strengths in favor of positive rewards, others in favor of negative rewards. Acceptance of such recommendations is indicated by firing of MSNs corresponding with the reward behaviors, and these MSNs modulate the firing of dopaminergic neurons targeting the basal ganglia to make long-term changes to the weights of recently active synapses on other MSNs that have recently fired (Coward 2013).

#### Information release processes

Another key set of information mechanisms are those that release information across the cortex at each point in time (Coward 2013). These mechanisms implement releases of subsets of current sensory inputs to primary sensory cortices (attention behaviors), implement releases of subsets of current pyramidal neuron condition detections in one cortical area to other areas (cognitive behaviors), and implement releases of subsets of current pyramidal neuron activity in motor cortex areas to drive muscle movements (motor behaviors). These releases are behaviors that are selected by the basal ganglia on the basis of recommendations by cortical inputs, and the basal ganglia triggers the thalamus to implement selected releases.

Each condition defined on a pyramidal neuron is some subset of its inputs from other neurons. If the neurons in that subset are all firing, the condition is detected. When a pyramidal neuron detects one of its defined conditions, it indicates that detection by firing. Firing of a neuron generates an output action potential (or spike) that goes to all its synapses on targeted neurons. When a spike arrives at a synapse, it injects an electrical potential that peaks about 2 ms after arrival of the spike and decays away exponentially (Mason et al 1991). Hence unless two spikes from different sources arrive within less than ~ 5 ms, they will not reinforce each other significantly. The activity of a group of pyramidal neurons in one cortical region is typically scattered in time (Tateno and Robinson 2006), and the outputs from an active group are therefore unlikely to trigger firing in their targets. The thalamic activity in response to a release selection by the basal ganglia imposes a gamma-band (~ 40 Hz) modulation on the group of cortical neurons selected for release [Cruikshank SJ. (1999) Thalamocortical inputs trigger a propagating envelope of gamma-band activity in auditory cortex in vitro Exp Brain Res 126:160–174]. This modulation bunches output spikes from different neurons in time, and therefore makes firing of their targets much more likely. Hence the 40 Hz modulation on cortical outputs placed by the thalamus effectively releases those outputs to their targets [Coward 2013].

### Episodic memory at high level

Episodic memory is the ability to recall information about specific individual events in past experience. In general, the greater the degree of novelty in the original experience the more effective the recall. There are thus two parts to episodic memory: the recording of information during the original experience; and the subsequent recall of significant parts of that information. To use a specific example, suppose that a brain experiences a traffic accident and was later asked “What happened at your traffic accident?”.

#### Recording a memory

In any situation, a number of cortical columns detect conditions within the information derived from the situation. If the number of columns detecting conditions is below a threshold, the hippocampal system drives condition expansions in some columns. When there are such expansions, the hippocampal system records different groups of cortical columns that were active when the expansions were occurring. These records and the actual expansions are the information recorded about the experience. In the case of a traffic accident, the experience has some aspects that are different from any prior experience. These novel aspects result in expansions to conditions in columns in a number of cortical areas, plus recording of conditions corresponding with groups of currently active cortical conditions (not just the ones that expanded) by the hippocampal system.

#### Retrieving a memory

Retrieving a memory requires activation of a significant proportion of the cortical conditions that were active during the original experience. This activation is in the absence of the sensory inputs that were present in the original experience. When the words “What happened at your traffic accident?” are heard, cortical conditions are directly activated in the auditory cortices. The conditions directly activated by hearing each word have recommendation strengths in the basal ganglia in favor of indirect activation of conditions in visual areas on the basis of frequent past simultaneous activity. The indirect visual activations in response to different words (like “traffic” or “accident”) are separate populations of firing neurons. If the behavior of bringing the outputs from these separate populations into the same phase of gamma-band modulation is accepted, a population of visual conditions activated in the past when situations corresponding with combinations of the words (for example “traffic accident”) were seen. Acceptance of all these recommendations drives activation of a population of conditions that is a weighted average of past circumstances in which the words “traffic” and “accident” have been heard close together. This seed population will not have enough recommendation strength to drive a verbal description of the actual accident, but has recommendation strength in favor of indirect activation of other conditions on the basis of past simultaneous activity during a period of receptive field expansion. The structure of the sentence results in detection of complex auditory conditions with recommendation strength in favor of accepting that type of recommendation. A secondary population is therefore activated that will have greater resemblance to the population active at the time of the specific traffic accident, much more likely to have sufficient recommendation strength in favor of a verbal description.

### Episodic memory at level of intermediate anatomical structures

Descriptions can be mapped from the highest level into the levels of more detailed anatomical structures. However, at this level the information content is much higher. Hence only small but key parts of the end-to-end recording and retrieval process will be described.

#### Recording a memory

A cortical receptive field is associated with a range of recommendation strengths. Receptive fields need to be changed to record new information, but any such changes risk jeopardizing the integrity of the recommendation strengths. There are a number of considerations that can reduce the risk. Firstly, after a change the field should still be detected in almost all the circumstances in which it was previously detected. This consideration is met if changes are generally expansions. Secondly, changes should be made only if necessary. Receptive fields recommend ranges of behaviors, with the fields in different areas recommending behaviors of somewhat different types. To achieve a high integrity behavior selection, there must be a reasonable number of ranges of behavioral recommendations available. In other words, at least a minimum number of receptive fields must be detected. This second consideration is therefore met if changes only occur when less than a minimum number of fields are already being detected. In other words, changes only occur if there is some degree of novelty. Thirdly, changes should be as small as possible. To meet this consideration, the inactive fields for which only a slight expansion will result in their activation must be identified and expansions limited to these fields. Finally, inactive fields that have changed in the past at the same time as some of the fields already being detected could be a better choice for the fields to change. To meet this consideration, groups of receptive fields that have tended to expand at the same times in the past must be recorded.

The primary role of the hippocampal system is to manage changes to cortical receptive fields in such a way that these considerations are satisfied (Coward and Gedeon 2015). The primary role of cortical columns is to generate the information needed by the hippocampal system. A cortical column is a group of pyramidal neurons with similar receptive fields (Tanaka 2003). There are generally five layers of pyramidal neurons in a column. Inputs from outside the column arrive mainly in layer IV. This input layer targets the middle layers II and III, and these middle layers target the output layers V and VI. Hence the complexity of the receptive fields increases somewhat going IV → II/III →V/VI. As a result, receptive fields in IV will be detected most often, those in II/III less often, and those in V/VI least often. Layer V receptive fields have the recommendation strengths in the basal ganglia. The functional value of this structure is that if there is no receptive field detection in the output layers but significant activity in the middle layers, the implication is that relatively slight receptive field expansions will produce outputs. The perirhinal, parahippocampal, and entorhinal cortices collect information about layer II/III activity from areas across the cortex, organized by groups of columns that have tended to expand their receptive fields at similar times in the past. This information is provided to the hippocampus proper, which determines the areas in the cortex where the degree of output activity is low, and generates signals to drive receptive field expansions in groups of columns across those areas that have tended to expand their receptive fields at similar times in the past as already active fields. These signals are decoded through the hippocampal cortices to drive receptive field expansions in selected columns with no output but significant layer II/III activity (Coward 2011). In this process, the receptive fields in the hippocampus proper and the hippocampal cortices also expand to update their receptive fields with information about the current group of receptive fields that are expanding at the same time.

In addition, in the hippocampal cortices, other receptive fields record information about the groups of currently active cortical receptive fields, including both those that are expanding and the others that are active without expansion. It is these fields that enable recall of episodic memories.

#### Recalling a memory

The striatum of the basal ganglia plays a role in the retrieval of declarative memories (Scimeca and Badre 2012). In the striatum, there are MSNs that correspond with the behavior of releasing selections of the activity in some cortical areas to the hippocampal cortices, and other MSNs that correspond with releasing hippocampal area activity to cortical areas. A seed population of receptive field detections is indirectly activated by hearing the words of the question. Columns in this seed population have connectivity on to striatal neurons associated with the behavior of releasing their activity to the hippocampal cortices. The basal ganglia constantly inhibits the thalamus. If the total inputs to those striatal neurons is sufficient, processes through the basal ganglia select the release behavior. This selection results in reduction of the inhibition of that part of the thalamus controlling releases from cortical areas into the hippocampal areas. The consequent releases drive activation of hippocampal neurons with receptive fields corresponding with groups of cortical columns active at the same time in the past during a period of receptive field expansion. Hence a population of columns is activated with receptive fields corresponding with groups of cortical columns that were active in past periods of receptive field expansion in which significant numbers of columns in the seed population were active.

Each of the hippocampal area columns is reciprocally connected with the cortical columns that form its receptive field, and also target MSNs corresponding with the behavior of releasing their outputs back to those cortical columns. Acceptance of this release behavior results in activation of a population of receptive fields that were active during a past period of receptive field expansion during which a significant proportion of the seed population receptive fields activated in response to “accident” and “traffic” were active. There is a reasonable chance that this population will have a significant resemblance to the population active during the original experience. Such a population would have recommendation strengths in favor of verbal description of the experience.

Note that the ability to generate such a description is not guaranteed. Successful retrieval will depend on the actual combination of recommendation strengths possessed by the seed population. This combination in turn depends on the degree of novelty in the original experience, and on any changes to recommendation strengths since the experience. For example, if the experience was recalled earlier, and the recall was followed by a positive reward, the recommendation strengths in favor of the steps in the recall will be increased.

Episodic memory recall of an experience is thus a sequence of information releases between cortical areas, ending with a population of active cortical receptive fields with significant resemblance to the population active during the original experience. Because memories are often recalled, the sequence is frequently used in the same order. The sequence will therefore tend to be recorded in the cerebellum and activated by the basal ganglia in response to detection of receptive fields within, for example, the structure of sentences requesting recalls. Note, however, that damage to the cerebellum will not result in complete loss of episodic recalls. The effect of such damage would be to revert to step-by-step selection of each behavior in the sequence by the basal ganglia.

#### Information releases

**Fig. 2:**
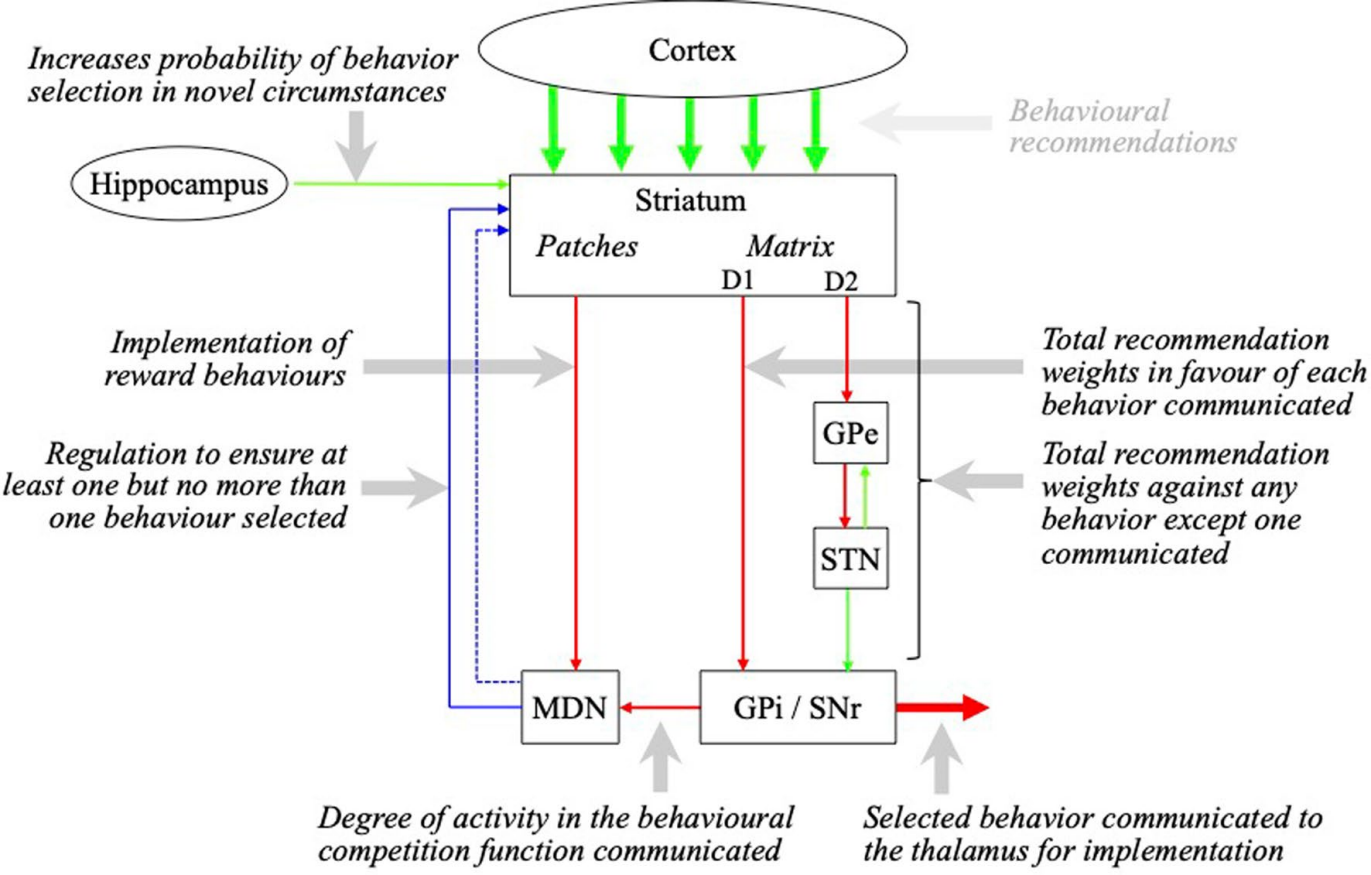
Circuitry of the basal ganglia (Coward 2013). Each input from the cortex is interpreted in the striatum as a range of recommendations in favor of different release behaviors. The release behaviors with the largest total weights are determined between the matrix of the striatum and the GPi/SNr. The GPi/SNr constantly inhibits the thalamus, and the selection of a behavior reduces the activity of the GPi/SNr neurons associated with the selected release behavior. The MDN regulates the striatum to perform two functions. One function is to ensure that in most circumstances at least one behavior is selected but not multiple incompatible behaviors. The second function is to implement reward behaviors recommended by cortical inputs to the patches of the striatum. Accepted reward behaviors change the recommendation weights that resulted in the selection of recent release behaviors. Some reward behaviors can change the recommendation weights in favor of other reward behaviors. Abbreviations: *GPe* globus pallidus external segment, *STN* subthalamic nucleus, *GPi/SNr* globus pallidus internal segment and substantia nigra reticulate, *MDN* midbrain dopamine neurons

**Fig. 3:**
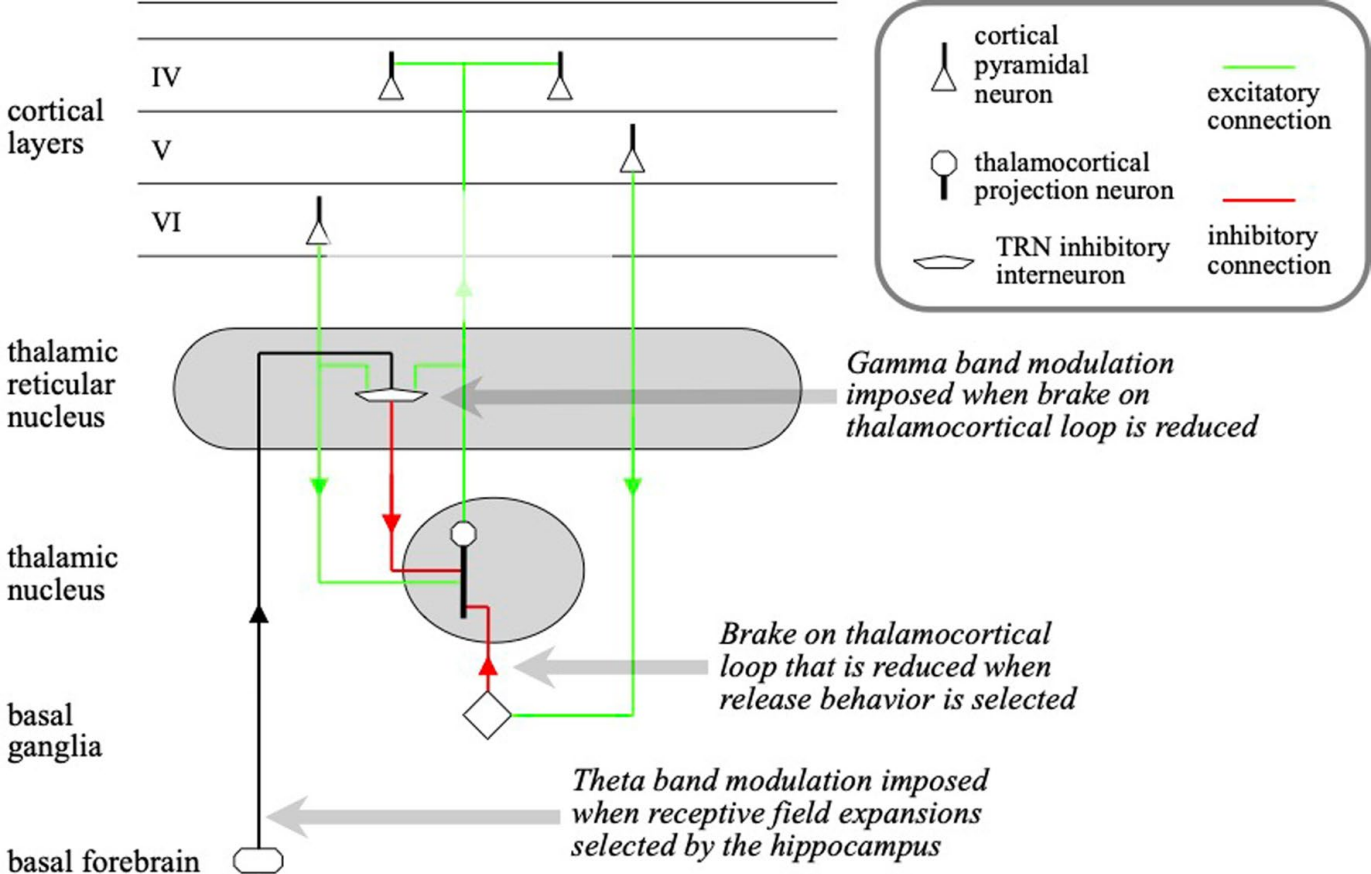
Thalamocortical connectivity and regulation by other anatomical structures (Coward 2013). Thalamocortical neurons in the thalamus excite pyramidal neurons in cortical layer IV. These layer IV neurons excite pyramidal neurons in layer VI (mainly via neurons in layers II/III not illustrated), and layer VI neurons target the thalamus. There is therefore a positive feedback loop between the cortex and the thalamus. Activity in the loop is kept in check by constant inhibitory input to the thalamus from the basal ganglia. Axons between the cortex and thalamus drop side branches on to inhibitory neurons in the thalamic reticular nucleus (TRN). If the basal ganglia selects a release behavior, the inhibition of the thalamic neurons corresponding with the release is reduced, and activity in the thalamocortical loop increases. This triggers firing of the TRN neurons. When these neurons fire, they generate a stream of spikes at the gamma-band frequency. This TRN output combined with the increased thalamocortical activity results in cortical activity producing spikes at the gamma-band frequency, out of phase with the TRN activity. If a receptive field expansion behavior is selected by the hippocampus, it is implemented by the basal forebrain imposing a theta-band modulation on top of the gamma band, bunching spikes even more to create a higher level of activity at which receptive field expansion will occur

The primary role of the thalamus is to release the outputs of groups of active cortical neurons within one cortical area to targets in other cortical areas. The thalamus also manages activity releases into and out of the cortex. The basal ganglia determines if there is sufficient cortical recommendation strength in favor of a release, and if so triggers the thalamus to make the release. The circuitry that performs these functions is illustrated in Figs. 2 and 3.

There are positive feedback loops between the thalamus and cortex. Cortical activity is constantly held in check by signals from the basal ganglia that inhibit the thalamus. Release behaviors are selected by the basal ganglia and communicated to the thalamus by a reduction in the steady inhibition of the thalamus. This reduction increases activity in the corresponding thalamocortical loop, but also results in a stream of inhibitory action potentials on that loop generated at the gamma-band frequency by TRN neurons. The combination of these two factors results in significant cortical outputs bunched together at points in gamma-band modulation out of phase with the TRM activity. These outputs therefore have a much stronger effect on their targets, effectively releasing them to those targets.

The gamma- and theta-band frequencies observed in the EEG thus reflect the management of releases of information into and out of the cortex and between cortical areas.

### Episodic memory at the level of neuron circuitry

The primary role of the hippocampal system is to manage changes to cortical receptive fields. This role requires definition of hippocampal system receptive fields corresponding with groups of cortical fields that expanded at the same time in the past. Indirect activations in support of episodic memory retrievals require definition of receptive fields corresponding with groups of cortical fields active during past periods of receptive field expansion, including both those that expanded and those that did not expand. Because of the similarity between the two types of receptive fields, resource economies can be achieved by defining them in the same anatomical structure. However, this must be done in such a way that there is no interference between the two functions. For example, if the same neurons were used for both functions, retrieval of an episodic memory would drive inappropriate receptive field expansions (Coward 2013).

**Fig. 4:**
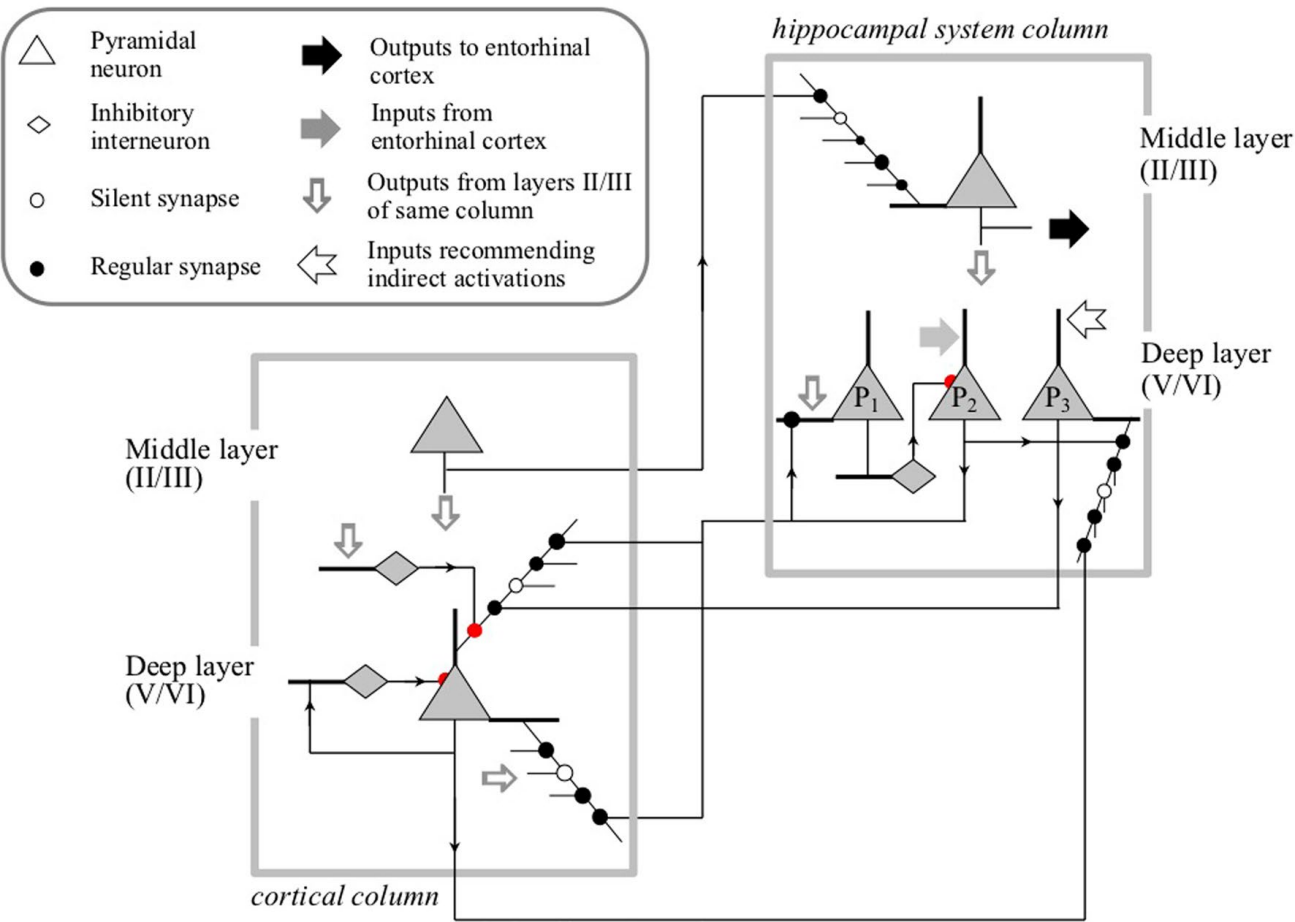
Detailed neuron circuitry between and within cortical and hippocampal columns. This circuitry supports receptive field change management and episodic memory retrieval in a way that economizes on resources by exploiting the information similarities between the two types of task, but avoids undesirable interference between them. For a full description, see Coward and Gedeon (2015)

As illustrated in Fig. 4, in the cortices associated with the hippocampal system, there are three populations of deep (V/VI) layer pyramidal neurons. As discussed in detail in Coward (2013), these three populations detect the receptive field of the column, drive receptive field expansions, and drive indirect activations in support of episodic memory retrievals, respectively.

## Conclusions

Dynamic system models of the brain are effective for modeling global brain activity such as EEG measurements. However, their dependence on a relatively unsophisticated approximation that observed phenomena reflect ensemble behavior and not that of individual neurons makes them incapable of modeling specific cognitive processes. There are no direct resemblances between brains and electronic computing systems. However, in brains the unique individual connectivity of neurons determines behavior, just as in computers the unique individual connectivity of transistors determines the operation of user features. The techniques used to understand the designs of computing systems therefore have some important lessons for how to go about understanding cognitive processes in terms of anatomy, physiology, and chemistry. In computing systems, architectural features such as the organization of information processing resources into modular hierarchies and a limited number of general types of information processes are key to establishing the hierarchies of description, with sophisticated management of the use of approximation, that are required to maintain design understanding. Natural selection pressures on brains have resulted in the presence of modular resource hierarchies and a limited number of general types of information processes.

Although in brains the modular hierarchies and information processes are qualitatively different from those in computing systems, their existence supports the creation of analogous hierarchies of description, which make possible an intuitively satisfying understanding of cognitive processes in terms of more detailed brain activity. A hierarchy of descriptions making it possible to understand recording and retrieval of episodic memories has been described. This hierarchy goes from cognitive descriptions, through major anatomical structures and detailed anatomical structures to descriptions in terms of neurons within individual anatomical structures. A similar approach should be possible for other cognitive phenomena including speech, consciousness, and self-awareness (Coward 2013; 2021).
